# Mutational spectrum of BRCA genes in Egyptian patients with breast cancer

**DOI:** 10.1038/s41598-025-09810-5

**Published:** 2025-07-18

**Authors:** Wafaa Elmetnawy, Heba Nader, Tamer ElNahas, Salwa Sabet, Heba Bassiony, Yasser ElNahass

**Affiliations:** 1 Department of Clinical Oncology, Faculty of Medicine, Cairo University, Cairo, Egypt; 2 Department of Zoology, Faculty of Science, Cairo University, Cairo, Egypt; 3 Department of Clinical Pathology, National Cancer Institute, Cairo University, Cairo, Egypt

**Keywords:** *BRCA1/2*, Breast cancer, Family history, HR+, HR−, TNBC, CNV, AMP, ACMG, Biotechnology, Cancer, Genetics, Molecular biology, Biomarkers, Diseases, Health care, Medical research, Molecular medicine, Oncology, Risk factors

## Abstract

The rising incidence of breast cancer (BC) among Egyptian females with a mortality rate of 11% and younger age at diagnosis implied the study of the interplay of *BRCA* gene variants with other BC risk factors. The study enrolled 500 BC Egyptian females with a mean age of 47.29 ± 13.26 years for whom *BRCA*1/2 testing was offered. A history of BC and/or other related cancer was recorded for all patients. Peripheral blood samples were obtained for genomic DNA extraction in view of germline *BRCA* gene testing on the MiSeq platform. A positive family history was reported in 352 patients (70.4%). Patients with hormone receptor–positive (HR+) BC constituted 195 cases (39%) cases, while 305 patients (61%) had hormone receptor–negative (HR−) BC. Among the HR− group, 268 patients (53%) had triple-negative BC (TNBC), and 37 patients had low estrogen receptor (ER) (1–10%) and/ or low progesterone receptor (PR) expression with HER2 negative status. Patients with HER2-positive BC were excluded from the enrollment and directed to specific targeted therapy. Variants were classified according to the American College of Medical Genetics (ACMG) and the Association for Molecular Pathology (AMP) criteria. Carriers of g*BRCA*1/2 PVs/LPVs were 58 patients (11.6%) of whom 34 (6.8%) had *BRCA*1 PVs/LPVs and 24 (4.8%) had *BRCA*2 PVs/LPVs. Patients with TNBC demonstrated a higher rate of g*BRCA*1/2 PVs/LPVs (17.5%). We recorded 55 PVs/LPVs in both genes, 44 single nucleotide variants (SNVs), and 11 copy number variations (CNVs). Three novel g*BRCA*1 LPVs; c.2791del, c.361G>T and c.4431dup and two novel g*BRCA*2 LPVs; g*BRCA*2 c.3139del and c.5690 dup were identified. Variants of uncertain significance (VUS) were found in 22 patients, of whom 13 (59%) had a positive family history of breast/ovarian cancer. Genomic testing for *BRCA*1/2 status as part of a routine BC diagnostic workup contributes to comprehensive BC risk assessment.

*Trial registration:* Egyptian National Cancer Institute IRB approval number: 2301-305-051. Date of registration: 24th Jan 2023.

## Introduction

Breast cancer (BC) is the most common cancer among Egyptian females, accounting for 18.9% of all malignancies and 38.8% of female cancers^1,2^. It is currently the second most common cause of Egyptian cancer mortality after hepatocellular carcinoma, with an estimated mortality rate of approximately 11% in 2020^3^. Although the incidence of BC in Egypt is lower than that globally reported^3^, the mortality rate of BC in Egypt is 19.1 deaths per 100,000 compared to 14.9 deaths per 100,000 in the U.S.^4^. The mean age of BC in Egyptian patients is 10 years younger than in Western countries^5^. The increasing morbidity and mortality associated with BC in Egypt represents a special challenge to health authorities^3^. According to the published national registry, approximately 80% of BC cases in Egypt are sporadic, and 18.7% are familial^6^. According to the American Cancer Society (ACS), about 5% to 10% of BC cases are thought to be hereditary^7^, where the involvement of genetic factors as the alterations of the highly penetrant BC-causing genes (*BRCA*1/2) deserves special evaluation^8,9^. Until recently, the volume of genetic testing among Egyptian women has been disproportionately low due to a complex interplay of social, economic, and cultural factors governing healthcare access.

Since their discovery, thousands of *BRCA*1/2 pathogenic/likely pathogenic variants PVs/LPVs have been identified^10,11^. Many have variable pathogenicity potential, conferring various effects on BRCA1/2 protein function^12,13^. The prevalence of *BRCA*1/2 PVs/LPVs varies between ethnic groups and geographical areas, leading to several comprehensive studies in different countries to determine the mutational profile of both *BRCA*1/ 2 genes^14,15^. A wide variation in their pathogenic potential in relation to other risk factors has been revealed. The landscape of *BRCA*1/2 PVs/LPVs accordingly became important for better identifying individuals at increased risk of the disease. The prevalence of germline *BRCA*1/2 (g*BRCA*1/2) PVs/LPVs in BC was reported earlier in other Middle East and North Africa (MENA) countries; 12.9% in Saudi Arabia, 15.1% in Morocco, 15.5% in Lebanon and 13.9% in Jordan^16–19^ and in other studies^15,20,21^. The presence of g*BRCA*1 PVs/LPVs is known to increase the risk of triple-negative BC (TNBC), a more aggressive subtype characterized by the absence of ER, PR, and HER2 expression, and thus do not respond to hormonal therapy or anti-HER2 targeted therapy, which are among the most attainable therapeutic options in Egypt^22^. In contrast, g*BRCA*2 PVs/LPVs are more commonly associated with estrogen receptor-positive BC^23–25^.

In Egypt, the socioeconomic and genetic variables underlying the biological factors associated with BC represent significant challenges. The landscape of *BRCA*1/2 disease-causing variants becomes mandatory to identify individuals at increased risk of the disease. The present study is an exploratory study of the BC population for whom *BRCA*1/2 testing was indicated according to the National Comprehensive Cancer Network (NCCN) guidelines version 1.2023.

### The present study aims

1. To assess the mutational landscape of g*BRCA*1/2 genes in a cohort of 500 female BC patients.
2. To highlight the correlation between BC subtypes and g*BRCA*1/2 PVs/LPVs in Egyptian patients.

## Methods

Relevant guidelines and regulations are carried out in all methods. All experimental protocols were performed according to the institutional ethical procedures and approved by the Institutional Review Board of the National Cancer Institute (IRB-NCI), Cairo University, Egypt (Date: 24thJan 2023/No. 2301-305-052) in accordance with the ethical standards of the Helsinki Declaration^26^. A written informed consent was obtained from all individual participants included in the study.

### Patient selection

Between January 2023 and January 2025, a total of 500 female BC patients with a mean age of 47.29 ± 13.26 years were examined by the Multidisciplinary team of the Oncology Department (MDT) of Cairo University. Immunohistochemistry (IHC) data, including tumor stage, Ki-67 index, histological and molecular subtypes, were obtained from pathology reports issued by the Pathology Department as part of routine diagnostic assessment. HR+ tumors were defined as those expressing ER and/or PR. Triple-negative tumors were negative for ER, PR, and HER2. HER2-positive tumors were excluded from the study. According to IHC data, patients with hormone receptor-positive (HR+) BC constituted 195 cases (39%), while 305 patients (61%) had hormone receptor–negative (HR−) BC. Among the HR− group, 268 patients (53%) had TNBC, and 37 patients had low estrogen receptor (ER) (1–10%) and/ or low progesterone receptor (PR) expression with HER2 negative status. For these patients, g*BRCA*1/2 testing was indicated according to the guidelines of the NCCN version 1.2023 and the Olympia study. Patients in this study were selected if they fulfilled at least one of the following criteria: early onset BC, defined as BC diagnosis at an age below 40 years^27,28^, a history of BC and/or other related cancer like ovarian or male BC cancer in one or more patient family members at age below 50 years, or BC with advanced tumor stage or high grade (Stage III-IV based on American Joint Committee on Cancer (AJCC)^29^. Patients with non-primary BC, incomplete clinical data, poor DNA quality, and those with confirmed HER2 positive phenotype were excluded. IHC data, including tumor stage, Ki-67 index, and histological type, were obtained from pathology reports issued by the Pathology Department as part of routine diagnostic assessment.

### DNA extraction and next generation sequencing

According to the manufacturer’s instructions, genomic DNA was extracted from peripheral blood via a QIAamp DNA Blood Mini Kit (Qiagen, Germany). DNA quality was checked and optimized using Qubit® 2.0 Fluorometer (Invitrogen, USA) for Polymerase chain reaction (PCR)-based library preparation with *BRCA* Pro Panel (Amoy Diagnostics Ltd, Xiamen, Fujian, China) according to manufacturer instructions. Libraries were sequenced on the MiSeq platform (Illumina), enabling the detection of germline SNVs/Indels and large germline rearrangements. This panel comprehensively covers all protein-coding regions, intron/exon boundaries, introns, and untranslated regions (UTRs) of the *BRCA*1 (NM_007294.4) and *BRCA*2 (NM_000059.4) genes. The amplified regions of *BRCA*1/2 were aligned to hg-19 human reference to detect deletions, insertions, and substitutions with all VCF files available. The analytical accuracy of the base calls was > 90%. The minimum coverage was 200× , the uniformity was 95%, and the accepted Q30 was over 85% bases. The variant allele frequency (VAF) of the detected PVs/LPVs was 30–60%. The coding exons and intron/exon junctions of *BRCA*1/2 were optimally covered and sequenced according to European Molecular Quality Network (EMQN) best practice guidelines for genetic testing^30^.

### Data analysis

All detected variants were interpreted according to the updated American College of Medical Genetics (ACMG) and the Association for Molecular Pathology (AMP) guidelines for *BRCA*1/2 genes^31^. Each variant was cross-checked against ClinVar and dbSNP databases, and novel variants were further validated for nomenclature accuracy and pathogenicity using in silico tools VarSome and Franklin. The categories of classification included pathogenic (class 5), likely pathogenic (class 4), variants of uncertain significance (VUS) (class 3), likely benign (class 2), and benign (class 1). Sequencing data were analyzed using the AmoyDx NGS Data Analysis System (ANDAS), a validated and CE–IVD–certified secondary and tertiary bioinformatics analysis pipeline. ANDAS performs automated demultiplexing, quality control, alignment to the human reference genome (GRCh37/hg19), variant calling, and annotation. It is specifically optimized for the AmoyDx *BRCA* panel to ensure high sensitivity and specificity in detecting *BRCA*1/2 pathogenic and likely pathogenic variants. Additional in silico prediction software and external curated databases, including BC information core (BIC), ClinVar, ClinGen, and Varsome, were used for better assignment of pathogenicity^32–37^.

### Statistical analysis

Statistical analysis was performed using IBM SPSS® Statistics version 22 (IBM® Corp., Armonk, NY, USA). Pearson’s chi-square (*χ*2) or Fisher’s exact tests were used to compare the clinicopathological data between patients with *BRCA*1 PVs/LPVs and patients with *BRCA*2 PVs/LPVs to estimate the association between hormonal status and patient age. A *p*-value ≤ 0.05 was considered statistically significant.

## Results

### Demographic and pathological characteristics of the study cohort

500 BC patients with invasive ductal carcinoma (IDC) as a prevalent histotype (88.8%) were enrolled in this study. Their mean age was 47.29 ± 13.26 years, and the median was 44 years (19–84). The clinical and pathological features of the whole cohort are summarized in Table 1.

**Table 1:** Clinicopathological features and demographic data of 500 BC patients.

Characteristics	Patient cohort n = 500	*BRCA1*/2 mutant type n = 58	*BRCA1*/2 wild type n = 442	*P*-value
Mean age in years (range)	47.29 ± 13.23 (19–84)	42.9 ± 11.6 (24–73)	48 ± 13.4 (19–84)	0.0028*
Age ≤ 50 years	313 (62.6%)	45 (9%)	268 (53.6%)	0.029*
Age > 50 years	187 (37.4%)	13 (2.6%)	174 (34.8%)	
**Hormonal status**				
TNBC	268 (53.6%)	41 (8.2%)	227 (45.4%)	0.028*
HR positive	195 (39%)	17 (3.4%)	178 (35.6%)	
**Family history**				
Positive	352 (70.4%)	53 (10.6%)	299 (59.8%)	0.001*
Negative	148 (29.6%)	5 (1%)	143 (28.6%)	
**Site**				
Left	288(57.6%)	30 (6%)	258 (51.6%)	0.148
Right	169(33.8%)	18 (3.6%)	151 (30.2%)	
Bilateral	43 (8.6%)	10 (2%)	33 (6.6%)	
**Histologic type**				
IDC	444(88.8%)	54 (10.8%)	390 (78%)	0.247
Other	56(11.2%)	4 (0.8%)	52 (10.4%)	
**Grade**				
I	2(0.4%)	0 (0%)	2 (0.4%)	0.776
II	139(27.8%)	15 (3%)	124 (24.8%)	
III	359(71.8%)	43 (8.6%)	316 (63.2%)	
**Ki67 index**				
≥ 20%	323(64.6%)	53 (10.6%)	270 (54%)	0.000*
< 20%	177(35.4%)	5 (1%)	172 (34.4%)	

IDC, invasive ductal carcinoma; TNBC, triple negative breast cancer; HR, hormone receptor.

*Significant.

There was a significant difference in age at diagnosis between *BRCA*1/2 PV/LPV carriers and non-carriers (42.9 ± 11.6 vs. 48.0 ± 13.4 years; *p* = 0.0028). A significant age difference was also observed between *BRCA*1 and *BRCA*2 PV/LPV carriers, with *BRCA*1 carriers being younger than *BRCA*2 carriers (39.1 ± 8.71 vs. 48.25 ± 12.79 years; *p* = 0.004). Germline *BRCA*1/2 PVs/LPVs were identified in 58 out of 500 BC patients (11.6%). Of these, 34 patients (6.8%) carried g*BRCA*1 PVs/LPVs, and 24 (4.8%) carried g*BRCA*2 PVs/LPVs. A history of BC and/or other related cancer in one or more patient family members at an age below 50 years was reported in 352 patients (70.4%). Among 268 patients with TNBC, 41 patients (15.3%) were carriers of g*BRCA*1/2 PVs/LPVs, of whom 28 patients (10.4%) with g*BRCA*1 PVs/LPVs and 13 patients (4.9%) with g*BRCA*2 PVs/LPVs (Tables 1 and 2). Patients with TNBC had higher *BRCA*1/2 PVs/LPVs rate (8.2%) than those with HR+ BC (3.4%). This patient category had the highest rates of proliferating tumor index (Ki67>20), family history of BC or related cancer, and earliest onset disease (age younger than 40).

**Table 2:** Biological features relevant to *BRCA1* vs. *BRCA2* mutational status.

Variable	*BRCA1* mutant n = 34	*BRCA2* mutant n = 24	*P*-value
Mean age in years (range)	39.1 ± 8.71 (26–63)	48.25 ± 12.79 (24–73)	0.004*
**Age (years)**			
≤ 40 years (n = 186)	21 (11.3%)	6 (3.2%)	0.005*
> 40 years (n = 314)	13 (4.1%)	18 (5.7%)	
**Hormonal status**			
TNBC (n = 268)	28 (10.4%)	14 (5.2%)	0.045*
HR+ ve (n = 195)	6 (3.1%)	10 (5.1%)	
**Ki67**			
High ≥ 20% (n = 323)	33 (10.2%)	21 (6.5%)	0.163
Low < 20% (n = 177)	1 (0.6%)	3 (1.7%)	
**Family history**			
Positive (n = 352)	29 (8.2%)	24 (6.8%)	0.05*
Negative (n = 148)	5 (3.4%)	0 (0.0%)	

TNBC, triple negative BC; HR, hormone receptor.

*Significant.

### Distribution of *BRCA*1/2 variants

Based on the standards and guidelines for interpreting sequence variants^31^, we identified 55 *BRCA*1/2 PVs/LPVs (including 44 SNVs in addition to 11 CNVs), of which 44 were reported in the Clinvar database^37^, and 11 were considered novel variants.

**Fig. 1:**
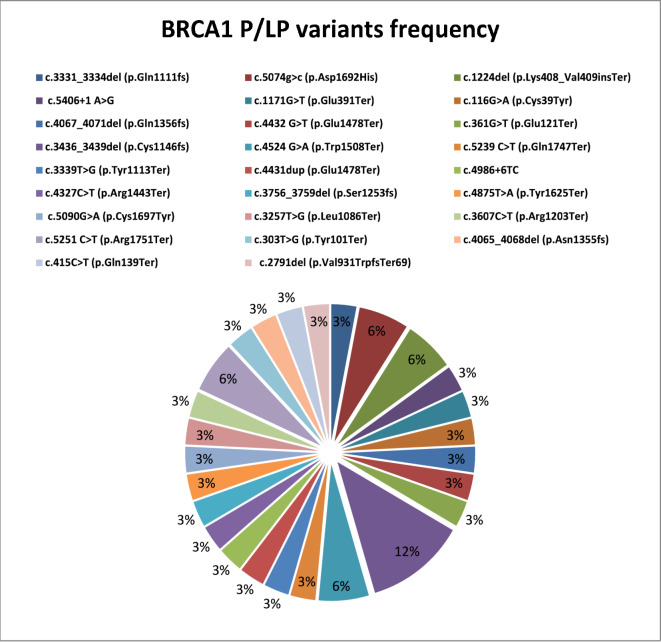
Distribution of *BRCA*1 PVs/LPVs in 500 BC patients.

**Fig. 2:**
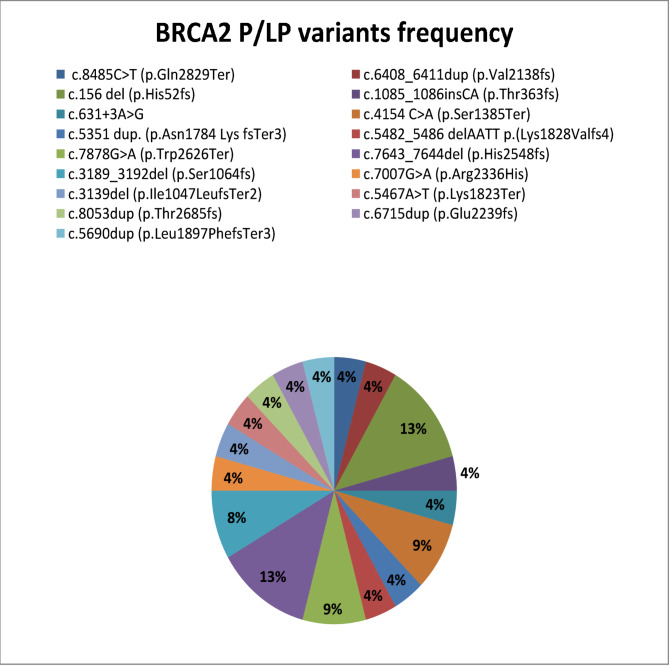
Distribution of *BRCA*2 PVs/LPVs in 500 BC patients.

Twenty-seven P/LP *BRCA*1 SNVs were detected, including 13 nonsense, 7 frameshifts, 5 missense, and 2 intronic variants **(**Fig. 1). Additionally, 17 *BRCA*2 P/LP SNVs were identified, including 11 frameshift, 3 nonsense, 2 missense, and 1 intronic variant (Fig. 2). Nonsense variants were prevalent in *BRCA*1 (13/27, 48.1%), whereas frameshift variants were more prevalent in *BRCA*2 (11/17, 64.7%). Patients carriers of g*BRCA*1 PVs/LPVs were characterized by younger age (< 40 years, 61.8%) and positive family history (85.3%).

Three novel LPVs g*BRCA*1 were identified, namely, c.361G>T (p.Glu121Ter) (patient A75), c.2791del (p.Val931TrpfsTer69) (patient M75) and c.4431dup (p.Glu1478Ter) (patient M338) located in exons 6, 10 and 11 respectively.

In *BRCA*2 gene, two novel frameshift LPVs were identified in exon 11; c.3139del (Ile1047LeufsTer2) (patient H418) and c.5690dup (p.Leu1897PhefsTer3) (patient M476) (Tables 3 and 4).

**Table 3:** *BRCA1* PVs/LPVs in 500 BC patients.

Patient ID	*BRCA1* Exon/Intron	HGVS nomenclature	Age	Hormonal status (HR)	Family history of cancer	Variant type	dbSNP rs	Clinical significance (ACMG)	Zygocity/VAF%	Database reported
M252	exon3	c.116G>A (p.Cys39Tyr)	37	Negative	Positive	SNV/missense	rs80357498	Pathogenic	Heterozygous/44%	Yes/eastern and western Europe
S433	exon6	c.303T>G (p.Tyr101Ter)	37	Negative	Positive	SNV/nonsense	rs80356936	Pathogenic	Heterozygous/51%	Yes/founder mutation in Africa
**A175**	**exon6**	**c.361G>T (p.Glu121Ter)**	**35**	**Negative**	**Positive**	**SNV/nonsense**	**Novel nonsense variant**	**Likely pathogenic**	**Heterozygous/50%**	**No/novel**
S459	exon6	c.415C>T (p.Gln139Ter)	43	Negative	Positive	SNV/nonsense	rs80357372	Pathogenic	Heterozygous/36%	Yes/western Europe
O68	exon9	c.1171G>T (p.Glu391Ter)	40	Negative	Positive	SNV/nonsense	rs562553169	Pathogenic	Heterozygous/50%	Yes/China
A33	exon10	c.1224del (p.Lys408_Val409insTer)	32	Negative	Positive	Deletion/nonesense	rs879255320	Pathogenic	Heterozygous/50%	Yes/Japan, Jordan
M77	exon10	c.1224del (p.Lys408_Val409insTer)	50	Positive	Positive	Deletion/nonesense	rs879255320	Pathogenic	Heterozygous/48%	Yes/Japan, Jordan
**M75**	**exon10**	**c.2791del (p.Val931TrpfsTer69)**	**63**	**Negative**	**Positive**	**Deletion/frameshift**	**Novel frameshift variant**	**Likely pathogenic**	**Heterozygous/46%**	**No/novel**
W399	exon10	c.3257T>G (p.Leu1086Ter)	35	Negative	Positive	SNV/nonsense	rs80357006	Pathogenic	Heterozygous/51%	Yes/western Europe
F19	exon10	c.3331_3334del (p.Gln1111fs)	60	Negative	Positive	Deletion/frameshift	rs80357701	Pathogenic	Heterozygous/50%	Yes/Jordan, Lebanon, Tunisia, western Europe , Native Latin America
N279	exon10	c.3339T>G (p.Tyr1113Ter)	26	Negative	Negative	SNV/nonsense	rs80357421	Pathogenic	Heterozygous/42%	Yes/Australia
S204	exon10	c.3436_3439del (p.Cys1146fs)	42	Negative	Positive	Deletion/frameshift	rs397509067	Pathogenic	Heterozygous/56%	Yes/China, Slovenia, Lebanon
H219	exon10	c.3436_3439del (p.Cys1146fs)	31	Negative	Negative	Deletion/frameshift	rs397509067	Pathogenic	Heterozygous/37%	Yes/China, Slovenia, Lebanon
S271	exon10	c.3436_3439del (p.Cys1146fs)	38	Negative	Positive	Deletion/frameshift	rs397509067	Pathogenic	Heterozygous/53%	Yes/China, Slovenia, Lebanon
T315	exon10	c.3436_3439del (p.Cys1146fs)	34	Negative	Negative	Deletion/frameshift	rs397509067	Pathogenic	Heterozygous/53%	Yes/China, Slovenia, Lebanon
A401	exon10	c.3607C>T (p.Arg1203Ter)	41	Negative	Positive	SNV/nonsense	rs62625308	Pathogenic	Heterozygous/47%	Yes/Western & eastern Europe and Latin America
M368	exon10	c.3756_3759del (p.Ser1253fs)	57	Negative	Positive	Deletion/frameshift	rs80357868	Pathogenic	Heterozygous/44%	Yes/Asia, Africa, Greece, Italy
N434	exon11	c.4065_4068del (p.Asn1355fs)	42	Positive	Positive	Deletion/frameshift	rs80357508	Pathogenic	Heterozygous/48%	Yes /Europe, Ashkenazi, America and Africa
I123	exon 11	c.4067_4071del (p.Gln1356fs)	38	Positive	Positive	Deletion/frameshift	rs2154266021	Pathogenic	Heterozygous/50%	Yes/Europe
M363	exon11	c.4327C>T (p.Arg1443Ter)	46	Positive	Positive	SNV/nonsense	rs41293455	Pathogenic	Heterozygous/40%	Yes/white Americans
**M338**	**exon11**	**c.4431dup (p.Glu1478Ter)**	**43**	**Negative**	**Positive**	**Insertion/nonsense**	**Novel nonsense variant**	**Likely pathogenic**	**Heterozygous/45%**	**No/novel**
G150	exon11	c.4432 G>T (p.Glu1478Ter)	42	Negative	Positive	SNV/nonsense	rs876659878	Pathogenic	Heterozygous/48%	Yes/western Europe
N207	exon14	c.4524 G>A (p.Trp1508Ter)	40	Negative	Positive	SNV/nonsense	rs80356885	Pathogenic	Heterozygous/31%	Yes/native America, western Europe, Ashkenazi, Saudi Arabia
G339	exon14	c.4524 G>A (p.Trp1508Ter)	31	Positive	Positive	SNV/nonsense	rs80356885	Pathogenic	Heterozygous/41%	Yes/native America, western Europe, Ashkenazi, Saudi Arabia
I370	exon15	c.4875T>A (p.Tyr1625Ter)	31	Negative	Positive	SNV/nonsense	rs886040251	Pathogenic	Heterozygous/44%	Yes/Europe
M352	intron15	c.4986+6T>C	42	Negative	Positive	SNV/intron variant	rs80358086	Pathogenic	Heterozygous/45%	Yes/African, Latin American ,Europe
F24	exon16	c.5074G>C (p.Asp1692His)	32	Negative	Positive	SNV/missense	rs80187739	Pathogenic	Heterozygous/60%	Yes/Ashkenazi
M268	exon16	c.5074G>C (p.Asp1692His)	33	Negative	Positive	SNV/missense	rs80187739	Pathogenic	Heterozygous/54%	Yes/Ashkenazi
M389	exon17	c.5090G>A (p.Cys1697Tyr)	31	Positive	Positive	SNV/missense	rs397507241	Likely pathogenic	Heterozygous/39%	Yes/Sudan, Europe, China, India
I471	exon17	c.5095C>T (p.Arg1699Trp)	48	Positive	Positive	SNV/missense	rs55770810	Pathogenic	Heterozygous/35%	Yes/western Europe, India, Morroco, Saudi Arabia
H253	exon19	c.5239 C>T (p.Gln1747Ter)	35	Negative	Positive	SNV/nonsense	rs80357367	Pathogenic	Heterozygous/45%	yes/western Europe , Asia
A421	exon19	c.5251 C>T (p.Arg1751Ter)	30	Negative	Positive	SNV/missense	rs80357123	Pathogenic	Heterozygous/45%	Yes/western Europe, African America, Asia
A487	exon19	c.5251 C>T (p.Arg1751Ter)	30	Negative	Positive	SNV/missense	rs80357123	Pathogenic	Heterozygous/35%	Yes/western Europe, African America, Asia
I37	intron21	c.5406+1 G>A	32	Negative	Positive	SNV/splice donor	rs80358028	Pathogenic	Heterozygous/53%	Yes/Europe

HR, hormone receptor; SNV, single nucleotide variant.

Significant values are in bold.

**Table 4:** *BRCA2* PVs/LPVs in 500 BC patients.

Patient ID	*BRCA2* Exon/Intron	HGVS nomenclature	Age	Hormonal status (HR)	Family history	Variant type	db SNP rs	Clinical significance (ACMG)	Zygocity/VAF%	Database reported
S16	exon3	c.156 del (p.His52fs)	28	Negative	Positive	Deletion/frameshift	rs2072285986	Pathogenic	Heterozygous/42%	Yes/USA, Europe
S458	exon3	c.156 del (p.His52fs)	62	Negative	Positive	Deletion/frameshift	rs2072285986	Pathogenic	Heterozygous/47%	Yes/USA, Europe
N496	exon3	c.156 del (p.His52fs)	66	Positive	Positive	Deletion/frameshift	rs2072285986	Pathogenic	Heterozygous/45%	Yes/USA, Europe
M25	intron7	c.631+3A>G intron variant	56	Positive	Positive	SNV/intron variant	rs397507840	Likely pathogenic	Heterozygous/43%	Yes/Western Europe
A2	exon10	c.1085_1086insCA (p.Thr363fs)	52	Positive	Positive	Insertion/frameshift	RCV003234819.1	Likely pathogenic	Heterozygous/54%	Yes/Arab (Once reported in Kuwait)
**H418**	**exon11**	**c.3139del (p.Ile1047LeufsTer2)**	**73**	**Positive**	**Positive**	**Deletion/frameshift**	**Novel frameshift variant**	**Likely pathogenic**	**Heterozygous/47%**	**No/novel**
S278	exon11	c.3189_3192del (p.Ser1064fs)	44	Positive	Positive	Deletion/frameshift	rs80359374	Pathogenic	Heterozygous/51%	Yes/Greece, Macedonia, Turkey, Latin American, Caribbean
K379	exon11	c.3189_3192del (p.Ser1064fs)	33	Positive	Positive	Deletion/frameshift	rs80359374	Pathogenic	Heterozygous/41%	Yes/Greece, Macedonia, Turkey, Latin American, Caribbean
A111	exon11	c.4154 C>A (p.Ser1385Ter)	44	Negative	Positive	SNV/nonsense	rs886038101	Pathogenic	Heterozygous/41%	Yes/western Europe
N343	exon11	c.4154 C>A (p.Ser1385Ter)	40	Positive	Positive	SNV/nonsense	rs886038101	Pathogenic	Heterozygous/43%	Yes/western Europe
H112	exon11	c.5351dup (p.Asn1784fs)	51	Positive	Positive	Duplication/frameshift	rs80359507	Pathogenic	Heterozygous/60%	Yes/Macedonia, Slovenia, Korea
S435	exon11	c.5467A>T (p.Lys1823Ter)	69	Positive	Positive	SNV/nonsense	rs276174858	Pathogenic	Heterozygous/46%	Yes/China
N169	exon11	c.5482_5486del (p.Lys1828fs)	24	Negative	Positive	Deletion/frameshift	rs80359516	Pathogenic	Heterozygous/48%	Yes/western Europe and Portugal , Asia
**M476**	**exon11**	**c.5690dup (p.Leu1897PhefsTer3)**	**57**	**Positive**	**Positive**	**Duplication/frameshift**	**Novel frameshift variant**	**Likely Pathogenic**	**Heterozygous/39%**	**No/novel**
R14	exon11	c.6408_6411dup (p.Val2138fs)	44	Negative	Positive	Duplication/frameshift	rs1555284670	Pathogenic	Heterozygous/48%	Yes/Europe
S460	exon11	c.6715dup (p.Glu2239fs)	46	Positive	Positive	Duplication/frameshift	rs1555284822	Pathogenic	Heterozygous/47%	Yes/Europe
N286	exon13	c.7007G>A (p.Arg2336His)	47	Negative	Positive	SNV/missense	rs28897743	Pathogenic	Heterozygous/52%	Yes/Iran, Italy, Saudi Arabia, Greece, America, Canada, Europe
S257	exon16	c.7643_7644del (p.His2548fs)	50	Negative	Positive	Deletion/frameshift	rs886040724	Pathogenic	Heterozygous/50%	Yes/western Europe
N298	exon16	c.7643_7644del (p.His2548fs)	38	Negative	Positive	Deletion/frameshift	rs886040724	Pathogenic	Heterozygous/45%	Yes/western Europe
I481	exon16	c.7643_7644del (p.His2548fs)	54	Negative	Positive	Deletion/frameshift	rs886040724	Pathogenic	Heterozygous/44%	Yes/western Europe
H178	exon17	c.7878G>A (p.Trp2626Ter)	45	Negative	Positive	SNV/missense	rs80359013	Pathogenic	Heterozygous/50%	Yes/China
B179	exon17	c.7878G>A (p.Trp2626Ter)	43	Negative	Positive	SNV/missense	rs80359013	Pathogenic	Heterozygous/50%	Yes/China
S436	exon18	c.8053dup (p.Thr2685fs)	62	Positive	Positive	Duplication/frameshift	rs397507958	Pathogenic	Heterozygous/30%	Yes/western Europe
R1	exon19	c.8485C>T (p.Gln2829Ter)	30	Positive	Positive	SNV/nonsense	rs80359099	Pathogenic	Heterozygous/43%	Yes/Europe

HR, hormone receptor; SNV, single nucleotide variant.

Significant values are in bold.

### Copy number variations (CNVs)

Software-based copy number analysis identified 12 variants in the *BRCA*1/2 genes, of which 5 were previously reported in the literature^15,37–42^ (Table 5).

**Table 5:** *BRCA1* and *BRCA2* CNVs detected in 500 BC patients.

Patient ID	*BRCA1/2* CNV	Age (Mean age 46.38 years)	Hormonal status (HR)	Family history	Reported	References
M52	*BRCA1* Exon 2 duplication (RING Finger Domain)	43	Negative	Positive	VUS	^35,36^
R266	***BRCA1*** Exon 2 deletion (RING Finger Domain)	38	Negative	Negative	Pathogenic	^38,39,42^
D263	***BRCA1*** Loss of Exon 2–20	50	Negative	Negative	Not reported	
M363	***BRCA1*** Exon 5–6 gain (RING Finger domain/coiled coil domain)	46	Positive	Positive	Pathogenic	^15,40,41^
B296	***BRCA1*** Exon 6 gain (coiled coil Domain)	40	Negative	Positive	Not reported	
D307	***BRCA1*** Exon 14–16 gain (BRCT domain /transactivation domain)	51	Negative	Positive	Not reported	
H163	***BRCA1*** Exon 16 loss (c terminal region in transactivation domain)	50	Negative	Negative	Pathogenic	^37^ ^Clinvar^ ^VCV000216106.3^
R303	***BRCA1*** Exon 21 gain (BRCT domain)	60	Negative	Positive	Not reported	
N465	BRCA1 Exon 20–24 gain (BRCT domain/transactivation domain**)**	28	Positive	Positive	Not reported	
L48	BRCA2 Exon 2 deletion (BRC repeat domain)	52	Negative	Positive	Pathogenic	^37^ Clinvar RCV000112809.1
D90	BRCA2 Ex11 deletion (central region/*RAD51*-binding Domain)	33	Positive	Positive	Not reported	
I155	BRCA2 Exon 12 gain (*RAD51*-binding Domain)	78	Negative	Negative	Not reported	
M156	BRCA2 Exon 12 gain (*RAD51*-binding Domain)	34	Positive	Negative	Not reported	

CNV, copy number variation, HR, hormone receptor.

Five novel *BRCA1* CNVs involving losses or gains of crucial parts of the gene were detected, including loss of exons 2–20, gain of exon 6, gain of exons 14–16, gain of exon 21, and duplication of exons 20–24. Additionally, two novel *BRCA2* CNVs were detected; a frameshift variant due to exon 11 deletion and an in-frame insertion resulting from exon 12 gain. Both variants contribute to genomic instability, which may have implications for cancer development and progression.

### Variants of uncertain significance (VUS)

Nineteen VUS were recorded in 22 (4.4%) patients; 8 g*BRCA*1 and 14 g*BRCA*2 VUS (Table 6). Thirteen patients (59%) with VUS had a positive family history of BC.

**Table 6:** *BRCA1* and *BRCA2* variants of uncertain significance (VUS) in 500 BC patients.

Patient number	Gene	Exon/Intron	HGVS nomenclature	Age	Hormonal status (HR)	Family history	Variant type	dbSNP rs	Clinical significance ACMG	Zygocity/VAF%	Database reported
I485	*BRCA1*	10exon	c.923G>C (p.Ser308Ter)	36	Positive	Positive	SNV/missense	rs561998108	Uncertain significance	Heterozygous/45%	Yes
N464	*BRCA1*	exon 11	c.1243G>A (p.Val415Ile)	57	Positive	Negative	SNV/missense	rs587782770	Uncertain significance	Heterozygous/37%	Yes
S447	*BRCA1*	10exon	c.2086A>G (p.Thr696Ala)	60	Negative	Negative	SNV/missense	rs80357441	Uncertain significance	Heterozygous/45%	Yes
S431	*BRCA1*	exon10	c. 3587C>T (p.Thr1196Ile)	69	Negative	Negative	SNV/missense	rs80356944	Uncertain significance	Heterozygous/49%	Yes
H439	*BRCA1*	exon10	c. 3587C>T (p.Thr1196Ile)	52	Positive	Positive	SNV/missense	rs80356944	Uncertain significance	Heterozygous/44%	Yes
H462	*BRCA1*	11exon	c.3836C>T (p.Ala1279Val)	56	Negative	Positive	SNV/missense	rs1555586967	Uncertain significance	Heterozygous/40%	Yes
W17	*BRCA1*	exon 14	c.4598A>T (p.Asp1533Val)	42	Negative	Positive	SNV/missense	rs2551470683	Uncertain significance	Heterozygous/45%	Yes
S491	*BRCA1*	exon 23 (3'UTR)	c.*381_*389delins27	60	Negative	Negative	indel/3 prime UTR Non Coding Transcript Variant	rs2152399492	Uncertain significance	Heterozygous/41%	Yes
M419	*BRCA2*	exon7	c.601C>T (p.Pro201Ser)	35	Positive	Positive	SNV/missense	rs1555281093	Uncertain significance	Heterozygous/50%	Yes
N334	*BRCA2*	exon10	c.1282C>A (p.Leu428Ile(	31	Negative	Positive	SNV/missense	rs547590567	Uncertain significance	Heterozygous/45%	Yes
N465	*BRCA2*	exon10	c.1550A>G (p.Asn517Ser)	28	Positive	Positive	SNV/missense	rs80358439	Uncertain significance	Heterozygous/48%	Yes
A260	*BRCA2*	exon11	c.2396A>G (p.Lys799Arg)	64	Negative	Positive	SNV/missense	rs1555282656	Uncertain significance	Heterozygous/43%	Yes
H453	*BRCA2*	exon11	c.2554A>C (p.Asn852His)	44	Negative	Negative	SNV/missense	rs775531301	Uncertain significance	Heterozygous/50%	Yes
M389	*BRCA2*	exon11	c.6101G>A (p.Arg2034His)	31	Positive	Positive	SNV/missense	rs80358849	Uncertain significance	Heterozygous/46%	Yes
A332	*BRCA2*	exon18	c.8141A>G (p.Gln2714Arg(	63	Positive	Positive	SNV/missense	rs80359059	Uncertain significance	Heterozygous/45%	Yes
N380	*BRCA2*	exon19	c.8435G>A (p.Gly2812Glu(	34	Positive	Negative	SNV/missense	rs80359091	Uncertain significance	Heterozygous/48%	Yes
S435	*BRCA2*	exon23	c.9104A>C (p.Tyr3035Ser)	69	Positive	Positive	SNV/missense	rs80359165	Uncertain significance	Heterozygous/48%	Yes
D263	*BRCA2*	exon24	c.9256+27T>A	50	Negative	Negative	Non Coding Transcript Variant	Novel itronic variant	Uncertain significance	Heterozygous/45%	No
G80	*BRCA2*	exon26	c.9586A>G (p.Lys3196Glu)	29	Negative	Negative	SNV/missense	rs80359228	Uncertain significance	Heterozygous/50%	Yes
R302	*BRCA2*	exon26	c.9586A>G (p.Lys3196 Glu)	31	Negative	Positive	SNV/missense	rs80359228	Uncertain significance	Heterozygous/55%	Yes
N334	*BRCA2*	exon26	c.9586A>G (p.Lys3196 Glu)	29	Negative	Negative	SNV/missense	rs80359228	Uncertain significance	Heterozygous/45%	Yes
N412	*BRCA2*	exon27	c. 10186A>T (p.Ser3396Arg)	43	Negative	Positive	SNV/missense	rs1555290062	Uncertain significance	Heterozygous/47%	Yes

HR, hormone receptor, VUS, variant of uncertain significance.

## Discussion

The present cohort represents a referral population from different Egyptian governorates, reflecting diverse personal and familial risk factors. The overall prevalence of g*BRCA*1/2 PVs/LPVs of 11.6% is not far from that reported in other MENA countries^16–19^. However, in China, a wide variation in the prevalence of g*BRCA*1/2 PVs/LPVs has been reported among patients with high‐risk BC, ranging from 8.3% to 17.6%^43,44^.

In our cohort, 8.2% of TNBC patients were carriers of g*BRCA*1/2 PVs/LPVs, whereas 9.3% were reported in Australia and 15.4% in the United States^45^. Consistent with other studies, g*BRCA*1 PVs/LPVs were more frequently detected in patients with TNBC- whose tumors are unresponsive to hormonal or HER2-targeted therapies—than g*BRCA*2 PVs/LPVs, representing 10.4% vs. 5.2%, respectively^22,46,47^. The patient category with g*BRCA*1 PVs/LPVs was characterized by younger age (< 40 years, 61.8%) and positive family history (85.3%), findings that should draw special attention to the need for genetic counseling programs.

The two reported novel g*BRCA*1 LPVs are located in exon 11 and are expected to confer a nonsense-mediated decay of RNA predisposing to differential breast or ovarian cancer risks and early onset BC^48^. Two patients had a strong family history of BC and the third developed contralateral BC.

Four patients (S204, H219, S271, T315) were carriers of the same pathogenic g*BRCA*1 variant, c.3436_3439del (p.Cys1146fs), previously described in Slovenia, China, and Lebanon^49–51^. These patients were originating from different unrelated families. However, it is common for recurrent mutations to be identified at elevated frequencies across different ethnic groups^52^.

Most pathogenic g*BRCA*2 variants were located in exon 11 and expected to disrupt the interaction of *BRCA*2 with other proteins, such as *RAD51* and *TP53*, which work together to mediate DNA damage repair^53^. Seventeen *BRCA*2 PVs/LPVs were previously reported in universal databases^54^. The *BRCA*2 novel mutations, c.3139del (Ile1047LeufsTer2) and c.5690dup (p.Leu1897PhefsTer3)*,* are located within the BCCR1 and BCCR2 regions, respectively. These regions are thought to mediate interactions with key transcription factors and DNA repair proteins, potentially disrupting crucial transcription factors and DNA repair proteins^55,56^. Notably, several of the *BRCA*1/2 PVs/ LPVs identified in our cohort have also been reported in other studies from the MENA region^15,17–21^.

In the literature, most large genomic rearrangements (LGRs) are pathogenic and are more commonly found within the *BRCA*1 gene than in *BRCA*2^57^. In our study, variants with abnormal copy numbers (CNVs) represented 18.6% of all variants, with more *BRCA*1 CNVs (12.9%) than *BRCA*2 CNVs (5.7%). A Dutch study reported 27% *BRCA*1 CNVs of all g*BRCA*1 variants^58^. Another study in China reported an incidence of 16.1% in *BRCA*1 CNVs^59^. *BRCA*1 CNVs involve gains or losses of crucial parts of the gene involving domains expected to affect genomic instability as the BRCT and c-terminal domains. They are associated with elevated BC risk, especially for patients with *BRCA*1 deletions^60^. Screening CNVs in BC patients who tested negative for g*BRCA*1/2 variants may indicate different therapeutic options, such as targeted therapy or immunotherapy.

The present study reported 22 (4.4%) patients with *BRCA*1 and *BRCA*2 VUS. The rate of VUS varies from 3.3% to 4.4% across different races and is the highest in the Asian population, with up to 15%^36,61–63^. The presence of VUS in 22 of our patients can be hard to ignore, primarily if a compelling personal and/or family history exists. Regularly updating variant databases within this context may help better define the clinical significance of VUS reclassification.

In the present study, 70% of patients had a positive family history of breast/ovarian cancer. In contrast, carriers of g*BRCA*1/2 PVs/LPVs represented only 11.6% of the BC cohort, which points to the stronger influential role of family history as BC predisposing^64,65^ and highlights the need to explore other genetic factors in patients who test negative for *BRCA*1/2 PVs/LPVs. Consistent with other studies, applying multigene panel testing of other BC predisposing genes with variable penetrance, such as *PALB2, PTEN, ATM, STK11,* and* CHEK*2, may provide a better comprehensive genomic picture of BC^66^.

The fact that approximately 26,000 new BC cases per year in Egypt are expected to reach 46,000 cases by 2050^3^ represents a challenge to health authorities regarding the underlying causes. It implies a need for more comprehensive BC screening programs that pinpoint early-onset BC cases. The routine application of genetic studies and implementation of counseling programs for this patient category is recommended.

### Limitations

This study has some limitations. Despite being one of the most extensive *BRCA*1/2 genetic screening studies among Egyptian BC patients, the sample size of 500 remains relatively small compared to Egypt’s large population of over 100 million. As a result, the sample size may not fully capture the prevalence of rare variants or represent the full genetic diversity of the broader Egyptian population.

## Conclusion

Our study ranks among the few studies in Egypt that explore the prevalence and spectrum of germline *BRCA*1/2 gene variants. Our findings point to the need for extended multigene profiling as part of routine BC risk assessment.

## Data Availability

VCF files are available from the corresponding author upon reasonable request. The data of current study are involved in the article.The patients’ clinicopathological data are presented in Tables 1 and 2, the detected SNPs and their accession numbers (rs) are illustrated in Tables 3, 4, 6) in the manuscript. The datasets and accession numbers generated and/or analysed during the current study are available in the [dbSNP] repository (https://www.ncbi.nlm.nih.gov/snp/) and [ClinVar] repository (https://www.ncbi.nlm.nih.gov/clinvar/)".

## Abbreviations

ACS: American cancer society
BC: Breast cancer
NCCN: National comprehensive cancer network
AJCC: American joint committee on cancer
ACMG: American college of medical genetics
HR: Hormone receptor
TNBC: Triple negative breast cancer
P/LP: Pathogenic/likely pathogenic
PVs/LPVs: Pathogenic variants/likely pathogenic variants
g *BRCA*: Germilne *BRCA*
SNVs: Single nucleotide variants
CNVs: Copy number variations
HGVS: Human genome variation society
AMP: Association for molecular pathology
BIC: Breast cancer information core
IDC: Invasive ductal carcinoma
MENA: Middle East and North Africa
BCCR: Breast cancer cluster region
ER: Estrogen receptor
PR: Progesterone receptor
HER2: Human epidermal growth factor receptor 2
LRGs: Large genomic rearrangements
EMQN: European molecular quality network
IRB: Institutional review board
